# Organizational Factors Associated With Retention of Certified Nursing Assistants and Direct Care Workers

**DOI:** 10.1093/geroni/igaa057.1238

**Published:** 2020-12-16

**Authors:** Katherine Kennedy, Robert Applebaum, John Bowblis, Jane Straker

**Affiliations:** Miami University, Oxford, Ohio, United States

## Abstract

Low retention of certified nursing assistants (CNAs) and direct care workers (DCWs) continues to be an unresolved problem for nursing homes (NH) and assisted living (AL) settings. While numerous studies have examined predictors of CNA retention in NHs, little attention has been paid to differences between settings of long-term care. To inform practice and policy related to growth in the AL industry, this study compares the predictors of CNA and DCW retention rates. The 2017 Ohio Biennial Survey of Long-Term Care Facilities provides facility-level information from 968 NHs (91% response rate) and 708 ALs (88% response rate). Using regression analysis, we compare the factors that predict retention rates among providers with complete data on retention and controls. The same covariates relating to structural and financial characteristics, as well as staffing, management, and a number of retention best practices are used. Average DCW and CNA retention rates were 66% and 61% in ALs and NHs, respectively, with some settings reporting very low (and even 0%) retention over a year. AL and NH providers rated the problem’s severity highest (6 out of 10) compared to retaining other licensed nurses. Similar and different predictors were found across financial, environmental, and managerial practices supporting retention. CNA and DCW retention strategies may not be equivalently meaningful between settings, given differing working environments, resources, and regulations. Aging services managers should be attuned to practices supporting retention in their industry.

